# Antithrombotic management in left sided- ablation and appendage device-closure procedures

**DOI:** 10.1016/j.ipej.2025.10.008

**Published:** 2025-10-21

**Authors:** Sheetal Vasundara Mathai, Fengwei Zou, Luigi Di Biase

**Affiliations:** Department of Cardiology, Montefiore Medical Center, Albert Einstein College of Medicine, 111 East, 210 Street, Bronx, NY, 10467, USA

**Keywords:** Anticoagulation, Atrial fibrillation, Catheter ablation, Left atrial appendage closure, Thromboembolism

## Abstract

Atrial fibrillation is the most prevalent sustained cardiac arrhythmia, and the therapeutic landscape for stroke prevention has expanded to include catheter ablation for rhythm control and left atrial appendage closure in patients unsuitable for long-term oral anticoagulation. Although minimally invasive, these interventions present complex thrombotic challenges. The left atrial and appendage environment is inherently prothrombotic due to structural remodeling, endothelial injury, blood stasis, and abnormal hemostasis-processes further amplified by procedural instrumentation such as transseptal puncture during left-sided ablation and structural interventions. Antithrombotic management requires a balance between thromboembolic prevention and bleeding risk. Pre-procedural imaging with transesophageal echocardiography or cardiac computed tomography remains essential for thrombus detection and risk stratification peri-procedurally. Direct oral anticoagulants provide pharmacokinetic advantages over vitamin K antagonists, including shorter half-lives and greater predictability, thereby facilitating periprocedural management. Post-procedural therapy must be individualized on the basis of stroke risk, device-related findings, and patient-specific characteristics. This review synthesizes current evidence on antithrombotic strategies in the setting of left-sided cardiac interventions.

## Highlights

• Continuous DOAC use is preferred during AF ablation for safe and effective periprocedural anticoagulation.
• Post-ablation anticoagulation should reflect patient risk and substrate, not just procedural success.
• Antithrombotic therapy after LAA closure must be tailored to procedure, patient, and device factors.

## 1 Introduction

Atrial fibrillation (AF) represents the most sustained common cardiac arrhythmia, with increasing incidence and prevalence in the United States (US) and globally. The projected prevalence of AF in the US is 12.1 million in 2030, and up to 215,000 incident case per year in Europe by 2030 [[1], [2], [3]]. The management landscape for AF has evolved substantially, encompassing both pharmacological approaches and an expanding array of catheter-based interventions, that includes catheter ablation for rhythm control and left atrial appendage closure (LAAC) for stroke prevention in patients unsuitable for long-term oral anticoagulation [2,4,5]. These minimally invasive procedures, while offering significant therapeutic benefits, introduce complex thrombotic and bleeding considerations that must be carefully managed throughout the periprocedural period. Transseptal puncture (TSP), a common gateway for left-sided cardiac interventions, has become increasingly prevalent with the growth of AF ablation, structural heart interventions, and LAAC procedures [4,[6], [7], [8]]. The optimization of antithrombotic strategies before, during, and after left-sided cardiac procedures requires balancing the competing risks of thromboembolism and hemorrhage. This narrative review synthesizes the current evidence and guideline recommendations for antithrombotic management across the spectrum of left atrial (LA) and LAAC procedures. We examine the pathophysiological basis for thrombotic risk, review pharmacological and imaging considerations, and provide practical, evidence-based strategies for periprocedural anticoagulation tailored to specific procedural contexts.

## 2 Pathophysiology of LA/LAA thrombus formation

**Fig. 1:**
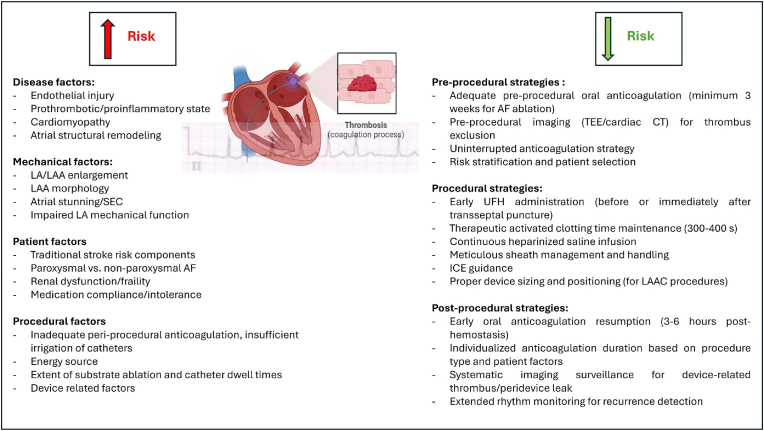
Balancing thrombotic risk in left atrial procedures: Pathophysiologic Drivers and evidence-based protective Strategies AF - atrial fibrillation, CT - computed tomography, ICE - Intracardiac echocardiography, LA - left atrium/left atrial, LAA - left atrial appendage, LAAC - left atrial appendage closure, SEC - Spontaneous echo contrast, TEE - transesophageal echocardiography, UFH - unfractionated heparin.

The prothrombotic and hypercoagulable milieu of the LA and left atrial appendage (LAA) arises from multiple pathophysiological mechanisms, largely encompassed by Virchow's triad: structural remodeling with endothelial injury, blood stasis, and abnormal hemostasis [9] [Fig. 1]. AF generates a prothrombotic and proinflammatory state in which inflammation and arrhythmia sustain and amplify one another. This involves increased inflammatory signaling, vascular endothelial growth factor (VEGF) upregulation, nitric oxide deficiency (as shown in animal models), and dysregulation of the renin–angiotensin–aldosterone system. Together these processes drive atrial remodeling and increase thrombogenicity [2,5,10,11].

Structurally, LA and LAA enlargement in AF and atrial flutter (AFL) exacerbate remodeling and promote stasis. Stroke risk rises with increasing LA size and impaired atrial strain on transthoracic echocardiography (TTE) or cardiac magnetic resonance imaging (MRI) [12,13]. Spontaneous echo contrast (SEC), a surrogate marker of stasis, independently predicts stroke in AF [14]. Elevated filling pressures-as seen after ablation, mitral stenosis, or restrictive cardiomyopathy, - further predispose to stasis and clot formation [9,15]. LAA morphology also plays a role, with complex trabeculations and certain shapes like the cauliflower or broccoli morphology conferring higher risk [7].

Therapeutic restoration of sinus rhythm leads to LA/LAA stunning and endothelial injury depending on the underlying method of rhythm control, pending recovery of atrial mechanical function that can predispose to thrombus formation [[15], [16], [17]]. The risk is further increased by pre-existing atrial myopathy, longer duration of arrhythmia, and larger atrial size, all of which prolong the duration and severity of stunning [15]. Distinct energy sources during ablation leads to silent cerebral lesions (SCLs) and TE through different mechanisms [18]. Resistive heating from radiofrequency (RF) lesions leads to direct endothelial denudation that promotes tissue factor release and subsequently thrombus formation both on the LA surface as well as catheter-blood interface. Other risk modifiers included prolonged catheter dwelling in the LA, longer time to therapeutic activated clotted time (ACT), extensive LA substrate ablation, and finally, need for periprocedural electrical cardioversions during the procedure are attributed risks [16,17]. Cryoballoon ablation, while less traumatic to endothelial tissue surface, causes temporary stasis inside the pulmonary veins during lesions formation. Large sheath exchanges may cause air embolism due to catheter exchanging through the same transseptal sheath [19]. In the era of pulsed field ablation, it is postulated that the non-thermal nature of electroporation would be less thrombogenic, opening up potentials of safer procedures and shorter duration of post-ablation anticoagulation need.

Procedural instrumentation of the LA/LAA contributes additional thrombotic risk. With the increasing use of minimally invasive valve interventions, atrial arrhythmia ablations, and LAAC, TSP has become common [6]. TSP introduces thrombotic risk through endothelial injury and exposure of the LA to foreign material (sheaths and catheters). Mechanical disruption exposes subendothelial tissue and activates the coagulation cascade, which is amplified by the low-flow environment of the LA and large surfaces for platelet adhesion [17,20]. Intracardiac echocardiography (ICE) studies show thrombus formation within 5–15 min of sheath entry into the LA, despite systemic anticoagulation at therapeutic levels [20]. Suboptimal sheath management, prolonged dwell times, and inadequate irrigation further heighten this risk.

Device-related thrombus (DRT) is another major concern after LAAC, with an incidence of 3–5 % in clinical trials and up to 7 % in real-world registries. Ischemic stroke occurs in up to 14 % of patients with DRT, underscoring the need for tailored antithrombotic strategies [7]. Mechanistically, incomplete reendothelialization of foreign device surfaces, particularly on the atrial aspect, creates a nidus for thrombosis in the setting of underlying stasis. Patient-related risk factors include prior stroke, larger LAA size, permanent AF, and history of LA thrombus. Procedural contributors include deep or off-axis implantation, incomplete ostial coverage, and oversized devices [7]. Inadequate closure causing peri-device leaks (PDLs) carries a thromboembolic risk comparable to that predicted by the patient's baseline CHA_2_DS_2_-VASc score, effectively negating the protective benefit of LAAC [9].

## 3 Imaging considerations in procedural AC management strategies

Pre-procedural imaging serves as the cornerstone for risk stratification and anticoagulation management in LA ablation and LAAC procedures. The LAA is the most common site for thrombus formation in patients with AF, and studies show LA thrombus prevalent in ∼2.7 % patients planned for catheter ablation for AF despite adequate OAC [5,21]. Risk factors included elevated stroke risk scores, non-paroxysmal AF, patients with cardiac amyloidosis, rheumatic heart disease or hypertrophic cardiomyopathy (HCM) necessitating pre-procedural imaging [21]. While 2D/3D transesophageal echocardiography (TEE) is considered the gold standard for peri-procedural imaging, computed tomography (CT) and ICE are emerging as an accurate and reliable alternatives [22,23]. In addition to thrombus detection, pre-procedural CT imaging facilitates decisions on anatomic feasibility, accurate device sizing based on measurements of the orifice, device landing zone and LAA length/depth, as well as peri-procedural planning including for TSP using double-oblique multiplanar views and volume-rendered 3D reconstruction [22]. Thrombus detection mandates procedural postponement and 3–6 weeks of therapeutic anticoagulation, with repeat imaging to ensure resolution [2,5]. Similarly, in patients undergoing left sided ablation for VT, contrast enhanced echocardiography to rule out LV thrombus is also warranted in patients with reduced LV ejection fraction [24].

Real-time imaging guidance during left atrial procedures employs multimodal approaches combining TEE, ICE, and fluoroscopy. ICE offers several advantages over TEE, including the ability to be performed under conscious sedation without the need for endotracheal intubation, reduced fluoroscopy exposure, shorter procedural times, early detection of thrombus formation or pericardial effusion, and improved outcomes [7,[25], [26], [27]]. ICE can be utilized for LAA thrombus screening prior to procedures [20]. For TSP, ICE enables real-time thrombus evaluation at the needle tip before entering the LA; if thrombus is identified, the apparatus can be retracted, flushed, and ACT reconfirmed prior to reattempting [25]. Prior to sheath removal, ICE can also reconfirm device positioning, rule out immediate device related thrombus, assess pericardial effusion. Post-procedurally, TTE to pericardial effusion and device embolization is warranted. Mid- and long-term post procedural imaging with TEE or coronary computed tomography angiography (CCTA) detects LAAC device related complications [28]. In a recent meta-analysis of 48 studies, CCTA demonstrated greater sensitivity in detecting PDL, however PDL detected by TEE was linked to adverse events over those detected by CCTA that lacked prognostic significance [29]. Similarly, DRT is associated with 4-to 5- fold increase in ischemic stroke risk and 2-fold increase in mortality [30]. Registry analyses have shown that up to one-third of DRT cases are detected beyond 6 months, supporting the rationale for re-imaging between 6 months and 1 year after LAAC [31,32]. Systematic follow-up beyond 3 months is warranted when early imaging detects abnormalities such as DRT, PDLs, incomplete endothelialization, or suspicious findings on routine imaging, with some experts recommending routine imaging at 1 year [7,28].

## 4 Pharmacological considerations

Vitamin K antagonists (VKAs), predominantly warfarin, were the principal medications in reducing TE risk, which is now currently limited to indications such as moderate to severe rheumatic mitral stenosis or mechanical heart valves, and to mitigate cost [2,5,33] Disadvantages that limit its use include a narrow therapeutic window based on international normalized ratios (INRs), frequent monitoring, more frequent drug interactions (mainly through CYP2C9), dietary restrictions, and low clinical safety [2]. Direct oral anticoagulants (DOACs) offer significant pharmacokinetic advantages over VKAs in periprocedural cardiac interventions and are the preferred agents in stroke prevention for AF. DOACs (apixaban, dabigatran, edoxaban, and rivaroxaban) achieve peak plasma levels within 2–4 h after oral administration and have shorter half-lives (5–17 h) compared to warfarin (7 days) and provide more predictable pharmacokinetics which enable safer periprocedural management with minimal interruption [33,34]. Choice of DOACs is influenced by concomitant medication use and their drug interactions, and patient factors such as age, frailty, renal or liver function, and other comorbidities [33]. Pertinent characteristics of both warfarin and DOACs are presented in Table 1 [2,5].

**Table 1:** Pharmacological considerations of anti-thrombotic therapies.

	Warfarin	Dabigatran	Apixaban	Rivaroxaban	Edoxaban
Mechanism of action	VKORC1 inhibitor	Direct thrombin inhibitor	Factor Xa inhibitor	Factor Xa inhibitor	Factor Xa inhibitor
Bioavailability	∼100 %	7 %	∼50 %	66 % fasting, 80–100 % with food	∼62 %
Time to peak effect	72–96 h	1h 2h with food	3–4 h	2–4 h	1–2 h
Half life	7 d	12–17 h	5–9 h	8–15 h	10–14 h
Excretion	92 % renal	80 % renal	27 % renal	66 % liver, 33 % renal	50 % renal
Standard dose	Target INR 2–3, TTR >70 % optimal	150 mg BID	5 mg BID	20 mg daily **with biggest meal**	60 mg daily
Reduced dose if criteria met		110 mg BID (EU([1])) ≥ 80 years, verapamil use Also consider when: age 75–80, CrCl 30–50, increased bleeding risk, GI issues 75 mg BID (USA) CrCl 15-30	2.5 mg BID: 2/3 criteria- age ≥80, actual body weight <60, serum Cr > 1.5 mg/dL ESRD on HD: no dose adjustment unless above criteria met	15 mg daily: CrCl 15–49 mL/min **with biggest meal**	30 mg daily: CrCl 15–50 mL/min, or weight ≤60 Kg
Reversal agent	Vitamin K + 4F-PCC > FFP + vitamin K	Idarucizumab, 4F- PCC	Andexanet alfa, 4F- PCC	Andexanet alfa, 4F- PCC	4F-PCC, Andexanet alfa

**4F-PCC** - 4-Factor Prothrombin Complex Concentrate, **BID** - twice daily, **Cr** – Creatinine, C**rCl** - Creatinine Clearance (mL/min), **ESRD** - End-Stage Renal Disease, **EU** - European Union, **FFP** - Fresh Frozen Plasma, **GI** – Gastrointestinal, **HD** – Hemodialysis, **INR** - International Normalized Ratio, **PCC** - Prothrombin Complex Concentrate, **TTR** - Time in Therapeutic Range, **USA** - United States of America, **VKORC1** - Vitamin K epoxide reductase complex subunit 1.

## 5 Anti-thrombotic strategies in AF

Current guidelines recommend initiation of OAC in eligible patients with elevated stroke risk (≥2 %) regardless of the temporal pattern of AF [2,5,16,20] The 2023 ACC/AHA guidelines recommend initiation of OAC for patients with annual stroke risk ≥2 %, preserving sex-based cutoffs when using CHA_2_DS_2_-VASc (≥2 in men, ≥3 in women), which is the most validated score [2]. Newer risk scores such as GARFIELD-AF and ATRIA may modestly improve risk discrimination (c-index) compared with CHA_2_DS_2_-VASc, while accounting for additional risk factors such as smoking, renal disease, and dementia in specific populations. The GARFIELD-AF score also includes mortality and bleeding risk to facilitate more comprehensive discussions with patients [2]. By eliminating birth-assigned sex—an age-dependent risk modifier rather than a true risk factor—the ESC recommends use of the CHA_2_DS_2_-VA score ≥2 for OAC initiation [5]. Antiplatelet therapy (aspirin alone or in combination with clopidogrel) is not an alternative to OAC in AF patients for preventing thromboembolism [2,5]. Factors that indicate higher bleeding risk, such as previous bleeding episodes or use of concomitant drugs that increase bleeding risk, should be evaluated. Furthermore, in the absence of absolute contraindications to OAC, bleeding risk scores such as HAS-BLED, HEMORR2HAGES, or ATRIA should not determine OAC eligibility. Rather, these tools should aid in identifying modifiable bleeding risk factors such as hypertension, excess alcohol intake, medication review, and adherence issues to inform decision-making during follow-up [2,5,35].

Peri-procedural TE events, namely stroke and SCLs occur at an incidence rate of 0.1–0.8 % and 5 %-40 %, respectively [2,17,18,20,36]. Adequate anti-thrombotic strategies and proper catheter handling techniques are paramount in reducing preventing TE events peri-procedurally. In patients undergoing catheter ablation for AF, initiation at least 3 weeks prior to the procedure is indicated to prevent peri-procedural TE events [2,5,20]. Table 2 [2,5,16,33] summarizes an evidence-backed practical approach to peri-procedural anti-thrombotic management strategies for catheter ablation in AF.

**Table 2:** Evidence-based practical approach of anti-thrombotic management for catheter ablation for AF in clinical practice.

Management phase	Recommendation	Evidence (Class, LOE)
***Pre-procedural management***		
	• Initiation of OAC at least 3 weeks prior to catheter ablation • Catheter ablation on uninterrupted DOACs should be the preferred strategy for eligible patients (eg, patients without mechanical heart valves, thrombophilia, or other clear indications for VKAs) • For patients undergoing AF ablation and therapeutically anticoagulated with VKA (INR 2–3) catheter ablation should be performed on uninterrupted VKA • Institutional protocol based on consideration of factors such as DOAC agent, dosing regimen, use of protamine after sheath removal in timing of last dose of OAC should be developed	2023 ACC*/AHA I-A* *2024 ESC 1-C*
		2023 ACC*/AHA I-A* *2024 ESC I-A*
		2023 ACC*/AHA I-B-NR* *2024 ESC I-A*
		*2021 EHRA*
***Intra-procedural management***		
AC management	• Intraprocedural intravenous anticoagulation with heparin (or direct thrombin inhibitors in those with heparin allergies) should be administered after femoral venous access and before transseptal puncture to reduce the risk of TE events. • Continuation of AC infusion to maintain ACT between 300 and 400 s • Administration of protamine is reasonable before sheath removal to facilitate adequate hemostasis	*2017 HRS/EHRA 1-B-NR*
		*2021 EHRA2017 HRS/EHRA I-B-NR*
		*2017 HRS/EHRA IIa B-NR*
***Post-procedural management***		
OAC management	• OAC should be continued for at least 2–3 months post catheter ablation for AF during the blanking period • Longer term OAC should be dictated according to stroke risk CHA_2_DS_2_-VASc ≥2, and not based on success or failure of the catheter ablation • In patients with contraindications to long term DOAC or high risk of bleeding and TE events (eg, CHA_2_DS_2_-VASc score ≥2), LAAO can be considered as an alternative to OAC	2023 ACC*/AHA I-B-NR* *2024 ESC 1C*
		2023 ACC*/AHA I-B-NR* *2024 ESC 1C*
		*2024 ESC IIb -C;*2023 ACC*/AHA IIa-B-NR* 2023 ACC*/AHA IIb-B-R*

**AC** – Anticoagulation, **ACT** - Activated Clotting Time, **AF** - Atrial Fibrillation, **CHA2DS2-VASc** - Congestive heart failure, Hypertension, Age ≥75 years (2 points), Diabetes mellitus, Stroke (2 points), Vascular disease, Age 65–74 years, Sex category (female), **DOAC** - Direct Oral Anticoagulant, **INR** - International Normalized Ratio, **LAAO** - Left Atrial Appendage Occlusion, **LOE** - Level of Evidence, **OAC** - Oral Anticoagulation, **TE** – Thromboembolic, **VKA** - Vitamin K Antagonist.

### 5.1 Periprocedural management

Performance of catheter ablation for AF with an uninterrupted OAC regimen, whether VKA or DOAC (uninterrupted or minimally interrupted), is currently endorsed by society guidelines [2,5]. This is evidenced by a growing body of evidence that proved superiority of an uninterrupted VKA strategy over VKA interruption pre-procedure [37], to reduce peri-procedural TE events [38,39] without increasing the risk of major bleeding or pericardial effusion [39,40]. Similarly, multiple RCTs demonstrated that uninterrupted DOAC strategy was noninferior to uninterrupted VKAs [[40], [41], [42], [43], [44]]. Further a meta-analysis of these trials demonstrated a significantly reduced risk of major bleeding (∼55 % relative risk reduction) for patients on uninterrupted DOACs over VKAs with no difference in minor bleeding or cerebral lesions [45]. In comparing periprocedural DOAC approaches (uninterrupted, mildly interrupted and interrupted), a meta-analysis of 43 studies assessing the pooled weighted mean of events showed no difference in major bleeding or TE events, but a slight increase in overall bleeding events in the minimally interrupted (8.62 %; 95 % CI: 5.16–12.86) vs uninterrupted (6.33 %; 95 % CI: 3.65–9.68), and fully interrupted DOACs (3.53 %; 95 % CI: 2.11–5.29) strategy [46]. Another meta-analysis of 18 studies accounting for 6203 patients showed no difference in risk for symptomatic TE, but an uninterrupted DOAC strategy was associated with a significant reduction in rate of SCLs on brain MRI compared to minimally interrupted DOACs (OR: 0.44; 95 % CI: 0.23–0.83; P = 0.01) [47]. On comparing bleeding rates, a meta-analysis demonstrated no sign of lower bleeding rates with preprocedural DOAC interruption (on morning of procedure), with similar rate of adverse clinical events (major bleeding, thromboembolic events) when compared to uninterrupted DOAC strategy [48].

Other important considerations include timing of the last DOAC dose in the context of pharmacokinetics (once-daily agents such as rivaroxaban and edoxaban typically given the evening prior, versus twice-daily agents such as apixaban and dabigatran given the morning of the procedure), renal function, timing of heparin bolus relative to TSP, protamine administration, choice of manual compression versus vascular closure device (VCD) for site management, and the center's experience performing groin punctures under anticoagulation. These factors collectively influence the balance between hemorrhagic and thromboembolic risk from therapeutic circulating drug levels [20,33].

### 5.2 Intra-procedural management

Unfractionated heparin bolus dose followed by infusion should be administered before or immediately after transseptal puncture to achieve and maintain an activated clotting time (ACT) of 300–400 s, with most authors favoring administration before TSP [16,19,20,33,34]. Total heparin dose and the time to achieve therapeutic ACT are higher in DOAC-treated than in VKA-treated patients with uncertain clinical implications [49]. Hence, it may be reasonable to use the same target ACT levels in both groups [33]. ACT levels should be assessed at 10–15 min intervals until therapeutic levels are attained and then repeated at 15–30 min for the duration of the procedure. Heparinized saline should be infused continuously through each transseptal sheath to further reduce the risk of thrombi [16,20]. Heparin infusion can be discontinued once all catheters are removed from the LA, and the sheaths removed from the groin when the ACT is less than 200–250 s. Sheaths can also be removed during full dose AC using vascular closure devices or figure of 8 suture [16,50]. Administration of protamine to reverse heparin effect is reasonable after AF ablation [16,19].

### 5.3 Post-procedural management

The decision to continue oral anticoagulation (OAC) after atrial fibrillation (AF) ablation requires balancing TE and hemorrhagic risks. Evidence from observational studies has been mixed. A recent large retrospective cohort of 1821 post-ablation patients showed that discontinuation at 12 months increased thromboembolic risk (0.86 vs. 0.37/100 person-years) but lowered major bleeding (0.10 vs. 0.65/100 person-years). Subgroups with asymptomatic AF, reduced left ventricular ejection fraction (<60 %), or left atrial enlargement (≥45 mm) were at particularly elevated risk when OAC was stopped, underscoring the need for individualized assessment [51].

In contrast, several meta-analyses of cohort studies suggest that discontinuation of OAC after successful ablation does not significantly raise thromboembolic risk, even in patients with higher CHA_2_DS_2_-VASc scores, while consistently reducing major bleeding events [45,52,53]. More recently, randomized and prospective data have begun to clarify this issue. The ALONE-AF multicenter randomized trial, which enrolled 840 patients with mean CHA_2_DS_2_-VASc 2.1 ± 1 and no atrial arrhythmia recurrence for ≥1 year post-ablation, found that OAC discontinuation led to a lower incidence of the composite endpoint of stroke, systemic embolism, and major bleeding compared with continued direct oral anticoagulant therapy (0 % vs. 1.4 %), primarily driven by fewer bleeding events. Importantly, ischemic stroke rates did not differ (0.3 % vs. 0.8 %) [54]. These findings suggest that, in carefully selected patients with durable sinus rhythm confirmed by extended monitoring, OAC cessation may be reasonable with shared decision-making. Ongoing trials such as OAT (NCT01959425) and OCEAN (NCT02168829) will further define the safety of discontinuation in higher-risk populations (CHA_2_DS_2_-VASc ≥1). Current guidelines recommend resuming OAC 3–6 h after adequate hemostasis, and continuing for at least two to three months post-ablation, and basing long-term therapy on stroke risk (CHA_2_DS_2_-VASc≥2 in men, ≥3 in women) rather than procedural success [2,5,20]. In the absence of clinical symptoms, discontinuation of OAC on a case by case basis can be considered in males with CHA_2_DS_2_-VASc≤1 and females with CHA_2_DS_2_-VASc≤2 [20]. In patients undergoing left atrial appendage electrical isolation (LAAEI) beyond PVI, lifelong OAC is warranted regardless of CHA_2_DS_2_-VASc score with electromechanical disruption of LAA function due to associated elevated TE risk [19,55]. Patients who are deemed unsuitable for lifelong OAC following LAAEI should instead be considered for left atrial appendage occlusion (LAAO) [19]. Similarly, among patients with hypertrophic cardiomyopathy and AF, OAC is indicated regardless of their stroke risk score [5].

### 5.4 AC management in the era of pulse field ablation (PFA)

PFA, a nonthermal ablation strategy, induces electroporation of cardiomyocyte membranes and proffers the potential to improve safety and efficacy even further, with lower thrombotic and inflammatory potential, as compared to traditional thermal energy sources like RF or cryoablation [56,57]. Theoretically, by eliminating char formation, vascular injury and local tissue inflammatory response, PFA could potentially reduce the risk of peri-procedural stroke. Rates of TE events post-PFA vary from 0 to 0.6 % mainly attributed to the learning curve with procedural characteristics such as catheter type, sheath handling, procedural duration or complexity and patient factors such as compliance [[57], [58], [59]]. Similarly rates of microembolic signals (MELs) with carotid echocardiography and SCLs (0–36 %) were also lower than RFAs [18]. A multicenter propensity score-matched prospective series including 800 patients evaluated OAC management following PFA and RFA on uninterrupted OAC. At 15-month follow up, stroke risk and arrhythmia recurrence was significantly higher in RFA vs. PFA group. Further, discontinuation of OAC after 1 month following the PFA procedure was not associated with any stroke/TIA vs.16 events (8.8 %) were reported in the RFA cohort, suggesting possibility of early cessation of OAC in PFA patients following 1–2 month blanking period in the absence of AF recurrence [57]. These rates were lower than that reported in the MANIFEST-17K multicenter registry study (0.12 %) owing to improved sheath management with experience, maintenance of ACR at >300 s and q15-30 min monitoring [60]. PFA-induced hemolysis is another significant consideration that increases with the number of pulses in a dose-dependent manner, that can contribute to AKI and anemia. There is no specific recommendation to alter anticoagulation solely due to post-PFA hemolysis unless there is clinically significant bleeding or renal dysfunction; individualized assessment is advised [61,62].

### 5.5 Anti-thrombotic considerations in special populations

Evidence suggests the benefits of DOACs over warfarin remains in patients with reduced kidney function. In a recent network meta-analysis of from four major DOAC trials, standard-dose DOACs demonstrated significantly lower stroke/systemic embolism (SSE) risk compared to warfarin in patients with creatinine clearance (CrCl) < 87 mL/min (4.8 % decrease in hazard ratio per 10-mL/min CrCl decrease; p-interaction = 0.01), with no CrCl threshold where DOACs had higher bleeding risk than warfarin down to 25 mL/min [63]. Another network meta-analysis including 42 studies with 185,864 subjects with kidney failure on hemodialysis showed lower risk of major bleeding on DOAC versus VKA (HR 0.74; 95 % CI, 0.64–0.84), with no difference in efficacy, all-cause mortality or TE risk. Among DOACs, dabigatran and rivaroxaban were associated with fewer embolic events [64]. Currently guidelines endorse consideration of OAC in the first two to three months after AF ablation even in chronic kidney disease (CKD) patients. The decision to continue OAC after 2 months should be based on the TE/hemorrhagic risk [65]. For patients with kidney failure on dialysis, it might be reasonable to prescribe apixaban [19,66].

The incidence of SSE increases progressively with age, from 0.75 events/100 patient-years in patients with AF aged <65 years to 2.2 events/100 patient-years in those aged ≥75 years [67]. Similarly, the risk of intracranial hemorrhage (ICH) is similarly at least two-times higher in those aged ≥80 years, irrespective of OAC use [68]. Among studies evaluating safety and efficacy of DOACs in elderly patients, DOACs had similar or superior efficacy compared to VKA in reducing SSE, with comparable or lower rates of major bleeding and ICH. Apixaban had the best combination of efficacy and safety in the elderly population [[69], [70], [71]]. Accordingly, in elderly patients undergoing AF ablation, authors suggest uninterrupted OAC to reduce the risk of TE events [19]. Further, initiation of DOACs should be preferentially considered in treatment naïve elderly (≥75 years) patients without traditional indications for VKA [19]. Interestingly, the multicenter, randomized controlled trial FRAIL-AF that included 1330 frail patients ≥75 years with atrial fibrillation (mean age 83 years) found that switching from VKA to DOACs increased bleeding complications (HR 1.69, 95 % CI 1.23–2.32), without reducing embolic events (HR 1.26, 95 %CI 0.60–2.61) [72]. The trial was stopped early for futility, concluding that switching from VKA to NOAC in frail older patients was associated with more bleeding complications, despite study limitations [73]. This is now incorporated in the 2024 ESC guidelines, wherein for older adults with AF who have been on long-term VKA treatment, it may be reasonable to consider its continuation, particularly among those with well-controlled INRs and no adverse effects [5].

## 6 Anti-thrombotic management in LAAC

### 6.1 Antithrombotic regimens

Concerns regarding advanced age, OAC adherence, and unfavorable bleeding risk profile in elderly patients have highlighted the need for effective and safe nonpharmacologic therapy for stroke prevention. Transcatheter LAAC is an appropriate option for patients with non-valvular AF who have a high TE risk, a reasonable life expectancy (>1 year), and preserved quality of life, but who are poor candidates with major bleeding risk, or have contraindications to long-term OAC therapy [2,4,5,9]. To address the question of need for OAC after AF ablation, the OPTION trial evaluated safety and efficacy of LAAC as an alternative to OAC post-AF ablation in patients at moderate to high risk for stroke (mean CHA_2_DS_2_-VASc score 3.5 ± 1.3). LAAC was associated with a lower risk of non–procedure-related bleeding and was noninferior with respect to the composite end point of death from any cause, stroke, or systemic embolism at 36 months [74]. Intra-procedurally during LAAC, unfractionated heparin administered before or immediately after TSP, with a goal ACT of 300 s is recommended [19]. The STOP-CLOT trial (NCT05305612) is evaluating whether early (pre-puncture) versus late (post-puncture) UFH administration reduces ischemic complications without increasing bleeding risk in structural transseptal interventions to formulate an optimal protocol [75].

**Fig. 2:**
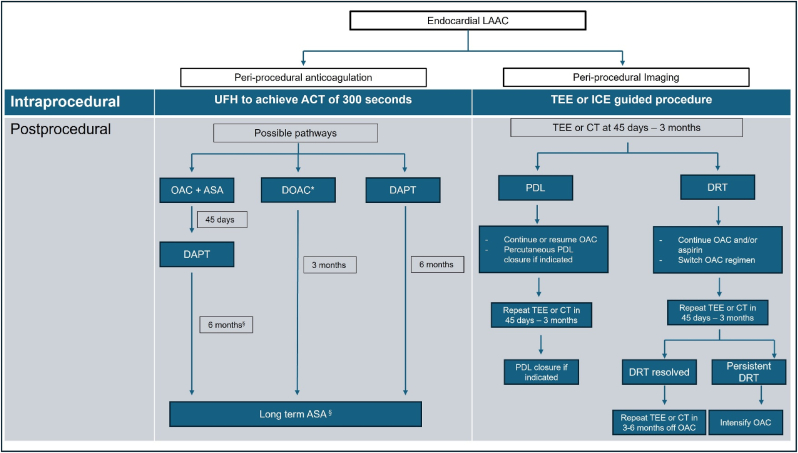
Antithrombotic Therapy and Imaging Surveillance After Left Atrial Appendage Closure **ACT** - Activated Clotting Time,**ASA** - Aspirin (Acetylsalicylic Acid), **CT** - Computed Tomography, **DAPT** - Dual Antiplatelet Therapy, **DOAC** - Direct Oral Anticoagulant, **DRT** - Device-Related Thrombus, **ICE** - Intracardiac Echocardiography, **LAAC** - Left Atrial Appendage Closure, **OAC** - Oral Anticoagulation, **PDL** - Peridevice Leak, **TEE** - Transesophageal Echocardiography, **UFH** - Unfractionated Heparin. ∗Can also consider half-dose DOAC plus aspirin (ASA). ^§^If no evidence of peridevice leak. ∗∗ OAC intensification – VKA (INR 2.5–3.5), low molecular weight heparin (LMWH), surgical excision if very large thrombus or OAC failure. If thrombus >15 mm, consider LMWH and repeat imaging in 2 weeks.

Anti-thrombotic strategies are currently warranted post-LAAC to mitigate the TE risk from DRT or PDL. Regimens include OAC (DOAC or VKA), dual anti-platelet therapy (DAPT), single antiplatelet therapy (SAPT) or a combination of agents for varying periods with the optimal strategy and duration still under investigation based on patient, procedural and device factors [Fig. 2] [Table 3] [74], [76], [77], [78], [79], [80], [81], [82], [83], [84], [85]. Current guidelines recommend post-procedural DOAC or DAPT [4,5,9]. At minimum the patient must be eligible for 4 weeks of anti-platelet therapy [9]. Results from the PROTECT-AF and PREVAIL trials proved the utility of warfarin plus aspirin for 45 days, followed by DAPT (aspirin and clopidogrel) for 6 months and SAPT with aspirin indefinitely. A pooled, patient-level analysis of the WATCHMAN trials by Price et al. demonstrated that while the overall rate of major bleeding was similar between LAAC and long-term warfarin therapy over 3 years, LAAC significantly reduced bleeding beyond the procedural period, especially after discontinuation of adjunctive pharmacotherapy [86]. Based on the results from PINNACLE-FLX study, DOAC plus aspirin for 45 days (6 weeks) post-procedurally was approved by FDA as an anti-thrombotic regimen post-LAAC [87]. Results from the AMULET-IDE study showed comparative efficacy and safety of 6 month-DAPT employed in Amulet arm, versus Watchman arm where 96 % received OAC on discharge [88]. The real world data from the EWOLUTION and NCDR LAAO registry, wherein patients discharged on DAPT had comparable adverse events as those discharged on OAC and aspirin [81,82]. In Europe, DAPT post-LAAC is now the favored regimen, which is also now approved by the FDA post-WATCHMAN-FLX as an alternate regimen [9].

**Table 3:** Summary of pivotal studies for antithrombotic Regimen post-LAAC.

Study	Device	N	Antithrombotic regimen	Outcomes
PROTECT-AF, 2009	Watchman	707	VKA + aspirin for 45 d, aspirin + clopidogrel for 6 months, aspirin indefinitely	**Primary endpoint:** CV death/stroke/SE 3.0 vs 4.9 events/100 pt-yrs (noninferior) **Bleeding:** Lower hemorrhagic stroke vs warfarin (1 vs 6) **DRT:** 3.74 % (pooled analysis) **Complications:** 7.4 vs 4.4 events/100 pt-yrs (mainly pericardial effusions)
PREVAIL, 2014	Watchman	407	VKA + aspirin for 45 d, aspirin + clopidogrel for 6 months, aspirin indefinitely	**Primary endpoint:** did not achieve non inferiority; secondary endpoint: Stroke/SE 7d-18mo: 2.53 % vs 2.00 % (noninferior) **Bleeding:** 10.8 % in device group **DRT:** 3.74 % (pooled analysis) **Complications:** Pericardial effusion 1.5 %, perforation 0.4 %, early safety 2.2 %
EWOLUTION Registry, 2019	Watchman	1020	Variable (∼60 % on DAPT, ∼27 % on OAC (VKA or DOAC ± aspirin), ∼7 % on SAPT)	**Primary endpoint:** Stroke 1.3/100 pt-yrs (83 % reduction vs expected) **Bleeding:** Major nonprocedural 2.7/100 pt-yrs (46 % reduction vs expected) **DRT:** 2.3 % **Complications:** Real-world data, lowest bleeding with early DAPT discontinuation
PRAGUE-17, 2019	Amulet Watchman/Watchman FLX	202 in LAAC group 114/67/5	DAPT for 3 months, aspirin alone if no thrombus on TEE	**Primary endpoint:** 3.5-yr HR 0.81 (non-inferior to DOAC) **Bleeding:** Major/nonmajor HR 0.81 (NS); 3.5-yr clinically relevant bleeding HR 0.55 (superior, p = 0.039) **DRT:** Not reported **LAA closure:** 96.8 % successful **Complications:** 9.4 % unsuccessful procedures
PINNACLE FLX, 2021	Watchman FLX	395	DOAC + aspirin for 45 d, DAPT for 6 months, aspirin indefinitely	**Primary endpoint:** 2-yr stroke/SE 3.4 % (1.7 % annualized) **Bleeding:** Major bleeding 10.1 % at 2 years **DRT:** No symptomatic DRT after 1 year **LAA closure:** 90 % at 1 year **Complications:** 0.5 % at 7 days, no pericardial effusions
Amulet IDE, 2021	Amulet WATCHMAN	903 896	DAPT for 45 days, aspirin indefinitely (75 % Amulet, 4.2 % Watchman) OAC for 45 d, DAPT for 6 months, aspirin indefinitely (21 % Amulet, 96 % Watchman)	**Primary endpoint:** Noninferior for safety/effectiveness **Bleeding:** Major bleeding 16.1 % vs 14.7 % at 3 years (p = 0.46) **DRT:** Lower with Amulet **LAA closure:** Superior with Amulet (PDL <3 mm: 90.5 % vs 78.3 %) **Complications:** 4.5 % vs 2.5 % (p = 0.02)
SWISS-APERO, 2021	Amulet Watchman/Watchman FLX	111 110	DAPT(∼60 %) or OAC for 3 months, aspirin daily	**Primary endpoint:** LAA patency 67.6 % vs 70 % (p = 0.71) **Bleeding:** Any bleeding 40.8 % vs 31.4 % at 13 months **DRT:** Definite/probable 2.4 % vs 3.8 % at 13 months **LAA closure:** Peridevice leak 13.7 % vs 27.5 % **Complications:** Major 9.0 % vs 2.7 % (p = 0.047)
NCDR LAAO Registry, 2022	Watchman	31994	Variable: VKA + ASA (36.9 %), DOAC + ASA (20.8 %), VKA alone (13.5 %), DOAC alone (12.3 %), DAPT (5.0 %)	**Primary endpoint:** Lower adjusted risk at 45d with VKA alone (HR 0.692) and DOAC alone (HR 0.731) vs VKA + ASA **Bleeding:** Included in composite endpoint **DRT:** Not specifically reported **Complications:** Only 12.2 % received full protocol; real-world practice varies widely
ADALA, 2024	Amulet Lambre Watchlan FLX	90 31/30 2/6 11/10	Low dose OAC 2.5 mg BID, aspirin daily vs. DAPT for 3 months, aspirin daily	**Primary endpoint:** 4.6 % vs 21.7 % (HR 0.19, 95 % CI 0.04–0.88, p = 0.02) **Bleeding:** Overall 4.6 % vs 28.3 % (p = 0.002); major 4.6 % vs 13.0 % **DRT:** 0 % vs 8.7 % (p = 0.045) **Complications:** 1 major bleeding during hospitalization (DAPT group)
OPTION, 2025	Watchman FLX	1600	OAC + aspirin for 90 d post-LAAC, aspirin to 12 months (LAAC performed with or after AF ablation)	**Primary endpoint:** Death/stroke/SE 5.3 % vs 5.8 % OAC (noninferior, p < 0.001) **Bleeding:** Non-procedural major/CRNM bleed 8.5 % vs 18.1 % (superior, p < 0.001) **DRT:** 1.9 % at 1-year **LAA closure:** 98.8 % success, seal in ∼80 % **Complications:** Major bleeding (including procedural) 3.9 % vs 5.0 % OAC, non-inferior, p < 0.001

**AF** - Atrial fibrillation, **ASA** - Aspirin (acetylsalicylic acid), **BID** - Twice daily, **CRNM** - Clinically relevant non-major (bleeding), **CV** – Cardiovascular, **d** – Days, **DAPT** - Dual antiplatelet therapy, **DOAC** - Direct oral anticoagulant, **DRT** - Device-related thrombus/thrombosis, **HR** - Hazard ratio, **LAA** - Left atrial appendage, **LAAC** - Left atrial appendage closure, **mo** – Months, **NCDR** - National Cardiovascular Data Registry, **NOAC** - Novel oral anticoagulant, **NS** - Not significant, **OAC** - Oral anticoagulation, **PDL** - Peridevice leak, **pt-yrs** - Patient-years, **SAPT** - Single antiplatelet therapy, **SE** - Systemic embolism, **TEE** - Transesophageal echocardiography, **VKA** - Vitamin K antagonist, **vs** – Versus, **yr/yrs** - Year/years.

Further, the ADALA study comparing low-dose apixaban (2.5 mg twice daily) against 3-month DAPT showed the former was associated with a reduction in primary composite outcome of major bleeding, thromboembolic events, and DRT, with reduction in combined major and minor bleeding with low dose apixaban compared to DAPT preferring evidence for a half dose DOAC regimen in high bleeding risk patients [85]. The OPTION trial further demonstrated feasibility of using an abbreviated regimen of DOAC plus aspirin for 90 days followed by SAPT with aspirin with low (1.9 %) DRT rates [74]. The role of SAPT or no antithrombotic therapy after LAAO in patients intolerant of short term OAC or DAPT has been evaluated in registry and observational studies showing comparable rates of DRT and stroke to conventional regimens [7,81,82]. Additionally, another ongoing trial named SIMPLAAFY (NCT06521463) compares two different monotherapy regimens — Aspirin and reduced dose DOAC — with DAPT in patients undergoing Watchman FLX Pro device implantation, while ASPIRIN-LAAO (NCT03821883) will evaluate outcomes following use of SAPT with aspirin with discontinuation of aspirin after 6 months. Ultimately, the choice and duration of antithrombotic therapy after LAAC should be individualized, taking into account the indication for LAAC, device-specific protocols studied, and patient-specific factors.

### 6.2 Management of DRT and PDL

Incidence of DRT in RCTs range from 1.7 to 4 %, with higher rates of DRT up to 18 % in real-world registry analysis with important clinical consequences [7,32,76,89]. Studies show association between DRT and increased risk for ischemic stroke, while also increasing risk for hemorrhagic stroke likely driven by need for reinitiation of OAC [32]. Independent predictors of DRT included permanent AF, history of TIA/stroke, vascular disease, larger LAA diameter, and a lower LVEF. Management options include initiation of OAC (DOAC or VKA with INR 2–3) with or without aspirin if patients were not on any OAC at the time of diagnosis for at least 8–12 weeks. In patients already taking OACs, ensuring compliance with intensification of regimen to INR goal 2.5–3.5, or switching to different DOAC classes should be considered and continued till imaging evidence of resolution [7,90]. Treatment is limited by high recurrence rates, varying DRT morphology, lack of consensus on optimal duration of treatment and patient factors such as candidacy for OAC and anticoagulation resistance [7].

PDLs, reported in 9–41 % of clinical trials and real-world registries, are associated with factors such as device malposition, inadequate compression, larger LAA dimensions, complex anatomy, and atrial remodeling in persistent AF. There is no consensus on the definition of a clinically significant PDL (<3 mm, >3 mm, or >5 mm) or on the optimal imaging modality for detection and localization [7,76]. Notably, even small leaks (<5 mm) have paradoxically been linked to increased stroke risk, potentially due to underuse of OAC in these patients [[91], [92], [93]]. A recent meta-analysis showed that the presence of any LAA patency was significantly associated with an almost 2-fold increased OR of thromboembolism (pooled OR: 1.87, 95 % CI: 1.08–3.24) [94]. Current evidence suggest treatment of PDLs >5 mm, either with OAC when feasible or with percutaneous closure techniques such as coils, plugs, additional occluders, or RF energy ablation to promote tissue remodeling and seal the defect [7,9,76].

## 7 Anti-thrombotic management for left-sided ablation procedures

### 7.1 Left atrial ablation for atrial tachycardia or left sided accessory pathways

While patients with atrial flutter have comparable TE risk as patients with AF [95], TE and SCLs were demonstrated post-catheter ablation for left sided accessory pathways (AP) and atrial tachycardias (AT) even in young healthy individuals. Routine pre-procedural anticoagulation is not recommended, although there is some variation in real-world practice [24,96]. Procedurally, there is usually only a single catheter with or without one long sheath in the left atrium or the left ventricle and short procedure time given the focal nature of ablation [24]. Further, patients undergoing AP ablation are usually younger, with few or no TE risk factors. Following TSP, 5000 to 15,000 U (or 90–200 U/kg) of unfractionated heparin is recommended followed by 1000 U/h during the procedure, targeting an ACT of 300 s, along with continuous flushing of the sheaths to avoid thrombus formation [24]. While routine post-interventional antithrombotic prescription is not recommended, decision must be individualized, with consideration of OAC in the setting of extensive ablation in the left atrium [19].

### 7.2 Left ventricular ablation for ventricular tachycardia (VT)

Anti-thrombotic strategies in VT ablation involve balancing TE risk (up to 2.7 % incidence) and hemorrhagic complications from access site and epicardial ablation when indicated [24,95]. In a recent German registry analysis, the incidence of stroke following VT ablation in both structural heart disease and idiopathic VT were 0.4 %, of which 75 % had their OAC held >1 day prior to the ablation [95]. Pre-procedural anticoagulation is not indicated, except in patients with a prior indication for OAC. Evidence now supports performing VT ablation on uninterrupted or minimally interrupted OAC [19]. Intra-procedurally, given TE risk profile similarity with AF ablation, therapeutic intravenous heparin and open irrigation RF ablation is recommended in patients with and without structural heart disease, which allows delivery of higher RF current before the catheter tip temperature reaches the point of coagulum formation. The epicardial sheath when used should regularly be aspirated during the procedure to reduce the risk of epicardial clot formation and tamponade [24]. Intraprocedural heparin administration is required before or immediately after TSP to maintain and activated clotting time longer than 250–350 s, along with continuous flushing [19,24,97]. While solely epicardial ablations do not warrant AC, in patients had already received heparin undergoing combined approach, administration of protamine before entering the epicardial space should be considered [97]. While there are no specific anti-thrombotic regimens specified post-procedurally, to mitigate TE risk, evidence now suggests a course of at least 4-week OAC (preferably DOAC) with extensive endocardial ablation, and antiplatelet for less extensive ablation for a limited period of time [19,97]. The STROKE-VT trial comparing post-procedural DOAC vs. Aspirin use following endocardial and/or epicardial left sided VT ablation showed reduced risk of TIA or stroke and SCLs in the DOAC arm, no significant difference in mortality and bleeding complications between the 2 groups [98]. In the absence of contraindications, post procedural OAC can be resumed/initiated 4–6 h [19].

## 8 Anti-thrombotic management for procedures needing TSP

The emergence of novel transseptal transcatheter interventions for mitral valve structural heart disease has fueled growing interest in optimizing transseptal puncture techniques. Current indications span percutaneous balloon mitral valvuloplasty (PBMV), transcatheter edge-to-edge mitral valve repair (TEER), transcatheter mitral valve implantation, paravalvular leak repair, which are usually approached with TSP [6]. In the landmark COAPT Trial, comparing TEER with the MitraClip™ device (Abbott) to guideline-directed medical therapy for the treatment of symptomatic severe secondary mitral regurgitation, the protocol mandated discontinuation of chronic OAC ≥3 days prior to the procedure, periprocedural UFH with a target ACT >250 s and a loading dose of clopidogrel before or immediately after the procedure [99]. Intra-procedurally, unfractionated heparin (2000 to 5000 U) is routinely administered before TSP, with additional dosing (total 200 U/kg) to achieve an activated clotting time (ACT) > 250 s after obtaining left atrial access [6,19]. Post-procedural antithrombotic strategies are highly procedure-specific: TEER typically requires 1–6 months of dual antiplatelet therapy (aspirin and clopidogrel) followed by aspirin alone for ≥12 months in patients without oral anticoagulation (OAC) indications, whereas transcatheter mitral valve implantation requires OAC with vitamin K antagonists for 3–6 months (target INR 2.5) to mitigate early thromboembolic risk during bioprosthetic valve endothelialization, potentially followed by lifelong low-dose aspirin [8,100]. In patients with indication for OAC, it should be continued post-procedure.

## 9 Conclusion

The expanding use of left-sided cardiac interventions for arrhythmia management and stroke prevention has necessitated a sophisticated understanding of periprocedural antithrombotic strategies. The optimal approach requires careful balance between thromboembolic and hemorrhagic risks, guided by procedure-specific considerations, patient-related factors, and emerging evidence from randomized trials and real-world registries. Uninterrupted DOACS have emerged as the preferred periprocedural strategy for atrial fibrillation ablation, offering pharmacokinetic advantages over VKAs. Critical to procedural safety is achieving and maintaining therapeutic activated clotting time levels, meticulous catheter handling, and real-time imaging guidance to detect early thrombotic complications. Post-ablation OAC strategies must be directed by individual TE risk, and not only success of ablation. For LAAC, post-procedural antithrombotic management requires individualization based on bleeding risk profile, device-specific considerations, and detection of complications such as DRT or PDLs. Emerging data supports abbreviated regimens and lower-intensity strategies in selected patients, though optimal approaches continue to evolve. Future research priorities include establishing intra-procedural anticoagulation protocols for left-sided ablations beyond atrial fibrillation, clarifying the thromboembolic risk profile with extensive left atrial substrate modification, defining optimal oral anticoagulation strategies in special populations, and refining risk stratification tools that integrate anatomical, functional, and procedural parameters. As catheter-based interventions continue to evolve, evidence-based antithrombotic management will remain essential to optimizing patient outcomes while minimizing procedural risks.

## Credit author statement

Sheetal Vasundara Mathai: Conceptualization; Data curation; Investigation; Methodology; Validation; Visualization; Roles/Writing – original draft; Writing – review & editing.

Fengwei Zou: Validation; Roles/Writing – original draft; Writing – review & editing.

Luigi Di Biase: Conceptualization; Data curation; Investigation; Methodology; Supervision; Validation; Visualization; Roles/Writing – original draft; Writing – review & editing.

## Declaration of competing interest

The authors declare that they have no known competing financial interests or personal relationships that could have appeared to influence the work reported in this paper.
